# Determining the Effect of the Picture Archiving and Communication System (PACS) on Different Dimensions of Users' Work

**DOI:** 10.1155/2022/4306714

**Published:** 2022-02-28

**Authors:** Mahdieh Montazeri, Reza Khajouei

**Affiliations:** Department of Health Information Sciences, Faculty of Management and Medical Information Sciences, Kerman University of Medical Sciences, Kerman, Iran

## Abstract

The impact of the picture archiving and communication system (PACS) on healthcare costs, information access, image quality, and user workflow has been well studied. However, there is insufficient evidence on the effect of this system on different dimensions of the users' work. The objective of this study was to evaluate the impact of the PACS on different dimensions of users' work (external communication, service quality, user intention to use the PACS, daily routine, and complaints on users) and to compare the opinions of different groups of users about the PACS. This study was performed on the PACS users (*n* = 72) at Kerman University of Medical Sciences, including radiologists, radiology staff, ward heads, and physicians. Data were collected using a questionnaire consisting of two parts: demographic information of the participants and 5-point Likert scale questions concerning the five dimensions of users' work. Data were analyzed using descriptive statistics, ANOVA, and Pearson's correlation coefficient statistical tests. The mean of scores given by the PACS users was 4.31 ± 0.86 for external communication, 4.18 ± 0.96 for user intention to use the PACS, 3.91 ± 0.7 for service quality, 3.16 ± 0.56 for daily routine, and 3.08 ± 1.05 for complaints on users. Radiologists and radiology staff had a more positive opinion about the PACS than other clinicians such as physicians (*P* < 0.01, CI = 95%). Factors such as user age (*P* < 0.01, CI = 95%), job (*P* < 0.001, CI = 95%), work experience (*P* < 0.001, CI = 95%), and PACS training method (*P*=0.037, CI = 95%) were related to the impact of the PACS on different dimensions of users' work. This study showed that the PACS has a positive effect on different dimensions of users' work, especially on external communication, user intention to use the system, and service quality. It is recommended to implement PACSs in medical centers to support users' work and to maintain and strengthen the capabilities and functions of radiology departments.

## 1. Introduction 

Due to the large number of medical images captured by different X-ray modalities, CT, MRI scanners, and sonography in health centers, storing and retrieving images are costly and time-consuming [1]. To address this problem, over the past decades, significant changes have taken place in medical imaging technology worldwide, leading to the digitalization of medical imaging devices [2]. These changes have led to the introduction and use of the picture archiving and communication system (PACS). PACS, along with other healthcare information systems, has changed hospitals' workflow and increased the efficiency of healthcare providers [3]. PACS is a computerized system used to collect, archive, process, communicate, and provide medical images and reports [4–6]. Studies have shown that this system reduces costs, prevents duplication of radiological images, increases image quality, reduces access time, and improves the quality of education, treatment, and security [7–10]. Studies have shown that younger radiologists are more interested in information technology-based systems such as PACS and radiology information system (RIS), and these systems can help them boost their careers in various ways [11]. A psychological study was conducted to assess the user acceptance of the PACS in the radiology ward of hospitals using the technology acceptance model (TAM). This study showed that the rate of technology acceptance was high [12]. Although many studies have examined the challenges of the PACS [3, 13–19] and the impact of this system on factors such as cost reduction, improved access, image quality [20, 21], and user workflow [11, 22], there is insufficient evidence on the effect of the PACS on different dimensions of the users' work. The objective of this study was to evaluate the impact of the PACS on different dimensions of users' work (external communication, service quality, user intention to use the PACS, daily routine, and complaints on users) and to compare the opinions of different groups of users about the PACS.

## 2. Materials and Methods

### 2.1. Research Setting

The study population comprised all PACS users in two academic hospitals of Kerman University of Medical Sciences, including radiologists, radiology staff, ward heads, and physicians. In this study, two hospitals having different PACSs and medical specialties were included in the study. Afzalipour Hospital is known for its internal medicine services and Shafa hospital for its cardiovascular services. At the time of this study, the PACSs had already been implemented in these two hospitals and are available in all clinical wards of these two hospitals. The PACS in Shafa has been implemented since 2017 and was purchased from MARCO, an Iranian company. The PACS in Afzalipour has been implemented since 2016 by Infinity, a Korean company.

### 2.2. Data Collection

The data collection tool was a questionnaire developed based on the previous studies in this field [23–25] and was completed by 72 participants. The questionnaire had two parts. The first part included eight questions about user characteristics and demographic information of the participants, and the second part included 25 questions concerning the five following dimensions: external communication (questions 9 to 11), service quality (questions 12 to 17), user intention to use the PACS (questions 18 and 19), daily routine (questions 20 to 26), and complaints on users (questions 27 to 33). A 5-point Likert scale (from very high to very low) was used to answer the questions of the second part.

### 2.3. Data Analysis

Data were analyzed by SPSS version 24. To analyze the data, question items were scored from 5 (very high) to 1 (very low). Analysis of variance (ANOVA) was used to assess the relationship between individual characteristics and the five dimensions of external communication, service quality, user intention to use the PACS, daily routine, and complaints on users. Pearson's correlation coefficient was used to measure the correlation between these dimensions. This study was approved by the Ethics Committee of Kerman University of Medical Sciences (code: IR.KMU.REC.1399.042).

## 3. Results

Demographic information of the participants is presented in Table 1. In this study, 78% of the participants were female, 47% had less than two years of experience with the PACS, and 51% had a mean level of computer knowledge. Among the users, 46% were physicians who worked with the PACS, and 65% had received group training about the use of the PACS.

**Figure 1:**
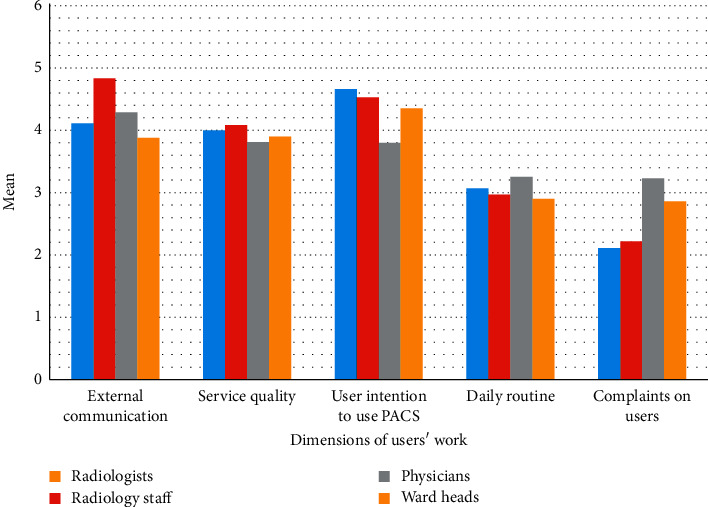
Mean scores of PACS impact per the dimensions of users' work.

Figure 1 shows the mean scores of the system impact per the dimensions of users' work. The highest scores were assigned by the radiology staff (4.07 ± 0.39) and radiologists (4.05 ± 0.56) and the lowest (3.48 ± 0.49) by the physicians to the dimensions studied in this study. The external communication dimension had the highest score (4.31 ± 0.86), and the complaints on users' dimension had the lowest score (3.08 ± 1.05). The mean scores of all dimensions were above 3.

Among three factors in the external communication dimension, the highest score was related to the factor “the impact of the PACS on accessibility and sharing of radiology data with other wards” (4.48, ±0.88). The mean score of all factors in this dimension was above 4.2. The highest score in the service quality dimension was related to “the impact of the PACS on management for services of the radiology” (4.06 ± 1.21). The mean scores of the factors related to this dimension were between 3.7 and 4.1. In the dimension of user intention to use the PACS, the highest score was related to the factor “I intend to use the PACS as much as possible” (4.45 ± 0.9). In the daily routine dimension, the highest score was related to the factor “the impact of the PACS on job satisfaction” (3.77 ± 1.07), and the lowest was related to “the impact of the PACS on the responsibilities and work requests of radiologists and radiology staff” (2.47 ± 1.06). The mean scores of all factors in this dimension were below 4. The highest score in the complaints on users' dimension was related to the factors “increasing the cooperation of the ICT department with the radiology department after the implementation of the PACS,” and the lowest score in this dimension was related to the factor “physicians and other clinical staff are less appreciative towards the PACS” (2.37 ± 1.41). Except for one factor, “physicians and other clinical staff are less appreciative towards the PACS,” the mean score of all factors in this dimension was higher than 3.

Table 1 shows the results of the ANOVA test to examine the relationship between user demographics and the impact of the PACS. The study variables had significant relationships with user age, work experience, occupation, and type of PACS training (*P* < 0.05, CI = 95%). According to this test result, the highest scores were given by users under 30 years old and the lowest score by users of 40 to 49 years old. By increasing the users' age and work experience, the mean of the score given to the effects of the PACS decreased. The highest score was given by the users having work experiences between 11 and 15 years and the lowest by the users with work experiences of 5–10 years. Individuals who received PACS training individually gave the highest scores, and those who received e-learning gave the lowest scores. Also, the scores of the service quality, user intention to use the PACS, daily routine, and complaints on users' dimensions were significantly related to the user age, work experience, and type of training (*P* < 0.05, CI = 95%) so that, by increasing the age and work experience, the mean of scores given to the mentioned dimensions declined. The users' computer knowledge was also related to the external communication dimension so that the mean of scores given by the users having an average knowledge or greater was almost twice as high as the mean of scores given by the beginners. Users with a work experience of lower than five years had the most, and people with work experiences between 11 and 15 years had the lowest desire to work with the PACS.

Pearson's correlation coefficient test was used to measure the correlation between the dimensions of external communication, service quality, user intention to use the PACS, daily routine, and complaints on users, the results of which are shown in Table 2. According to these results, the highest correlation was between the two dimensions of external communication and service quality (*r* = 0.631, *P* < 0.05) and the lowest correlation between the two dimensions of daily routine and service quality (*r* = 0.265, *P*=0.02).

## 4. Discussion

### 4.1. Principal Findings

This study showed that the PACS has a positive effect on different dimensions of users' work, so the participants gave a score of higher than 2.5 to all the dimensions on the 5-point Likert scale. Based on the results, PACS improves external communication, increases user intention to use the PACS, enhances service quality, streamlines daily routine, and reduces user complaints, respectively. The participants gave a mean score of approximately 4 out of 5 to the three dimensions of external communication, user intention to use the PACS, and service quality.

### 4.2. Comparison with the Results of Other Studies

The positive effect of the PACS on different dimensions of users' work that was shown in this study was in line with the results of previous studies [23, 26, 27]. The present study found that more than two-thirds of users believed that the PACS had improved external communication. Consistent with our findings, Hains et al. [28] examined the effect of the PACS on the physicians' work in the ICU and concluded that the implementation of the PACS improves physicians' efficiency and communication between the ICU and the radiology department. The reason is that electronic communication processes have changed the image viewing patterns and reduced physicians' visits to the radiology department to view radiology images [28]. Implementation of the PACS also improves communication between physicians and radiologists by facilitating joint decision-making [11, 29].

This study showed that the majority of the participants were inclined to use the PACS. This tendency could be due to the ease of use of the system [30, 31]. As stated in the technology acceptance studies in medical settings, the use of the PACS depends on its usefulness and ease of use [32–34]. It seems that embedding various tools and features in today's PACS interface has made this system useful and easy to use. The existence of multiple features in the system can meet users' expectations and result in service quality and user intention to use the PACS. However, other PACSs can have different results depending on their characteristics.

Consistent with the results of previous studies [26, 31], the results of our study showed that the implementation of the PACS improves service quality. This improvement can result from better management of radiology services and provision of better information for decision-making, by improving access to information, after the implementation of the PACS. In particular, according to the results of previous studies, PACS supports physicians' decision-making [21] and diagnosis [35] due to greater access to images and reports. Nevertheless, the results of a study conducted by Watkins [36] showed that the PACS can increase access to images but does not affect clinical decision-making. This difference could be related to the qualitative methodology with fewer participants used in this study.

This study showed that more than half of the users believed that the implementation of the PACS had supported the users in performing their daily routine. Congruent with our findings, Ayal and Seidman [37] showed that the PACS reduces the time required for daily routine and increases user satisfaction. It provides ubiquitous access to images, reduces the time of image recovery and clinical reports, enables effective planning to use radiology devices, facilitates teleconsultation, and provides assistive devices to support image recognition and improve hospital workflow. Another study [39] showed that the PACS improves daily routine by reducing image searching and interpretation time and speeding up the diagnoses.

Consistent with the results of a previous study [26], our findings showed that, after PACS implementation, complaints on users in the radiology department were decreased. Also, Trumm et al. [38] reported that replacing analog and film-based systems with the PACS reduced the problems of the radiology department and ultimately increased user satisfaction and reduced complaints on radiology staff.

### 4.3. Relationship between the Impacts of the PACS and Demographic Information of the Participants

The results of our study showed that factors such as user age, job, work experience, and PACS training method are related to the impact of the PACS on different dimensions of users' work.

According to the results, PACS had a positive effect on the work dimensions of all groups of PACS users. A previous study [39] showed that physicians believe that the PACS is more reliable than the analog system due to various features such as editing functionalities and the ability to make different changes in images and to compare previous and new images of the same patient. Physicians believed that the PACS improves the efficiency of the patient's follow-up process because of the ability to access images from multiple locations and easy consultation among different wards. According to the results of our study, the type of users' job affects their perception about the PACS. Among the main users of the system, radiologists and radiology staff had a more positive opinion about the PACS than other clinicians such as physicians. This may be due to the novelty of the PACS for physicians compared to radiologists and their unfamiliarity with this system. Consistent with this finding, Alalawi et al. [23] concluded that radiologists pay much attention to the strengths of the system than physicists and believe that the PACS improves their efficiency. Also, Buabbas et al. [31] showed that more than three-quarters of the radiologists found the PACS user-friendly and were positive about it.

By increasing the age and experience of participants in this study, their perspectives about service quality were more positive, and their intention to use the PACS decreased. This may be because users with a lower work experience used the PACS for most of their work period and could easily adapt to the system. Still, people with a higher work experience are resistant to using the PACS due to the long-term use of the previous system and need time to change their habits. Various studies have shown that users who had more experience with the PACS had a more positive view of the system [26, 40]. Previous studies have also shown that young people are more inclined to use information technology and applications [41, 42]. However, various studies have shown that improving the user interface and addressing system ease of use can increase the willingness of older adults to use these systems [43, 44].

One of the reasons that users in this study were positive about the PACS was that most of them were trained in the use of the PACS. In line with our results, other studies have shown that user training can contribute to the successful implementation of the PACS [19, 39]. Despite the usefulness of training, Bramblz et al. showed that this training does not always exist. For example, study participants expressed that “image contrast is not controllable” and “changing the darkness or brightness of the images is impossible,” while these features were present in the system, but users had not received training about them [21]. According to the findings of this study, PACS had the greatest impact on the work dimensions of users who received individual system training. Another study [39] showed the greater impact of individual training compared to other methods (group training, training through manuals, and electronic files) on user learning, use of system capabilities, and its successful implementation. Although training can help to identify a system's capabilities, the results of this study showed that all trained and untrained users had a favorable opinion about the impact of the PACS on various dimensions of their work.

In this study, the highest correlation was between the two dimensions of external communication and service quality. Previous studies [45, 46] also showed the positive effect of external communication on service quality. It is suggested that the authorities of medical centers that have no PACS put the hospital-wide implementation of the PACS on their agendas to increase service quality.

### 4.4. Limitations

The present study had three limitations. This study was conducted only on two types of PACSs implemented in two hospitals. Future studies can address other types of PACSs in several geographical areas and on a larger number of participants. Second, this study examined the impact of the PACS only on the five dimensions of users' work (external communication, user intention to use the PACS, service quality, daily routine, and complaints on users). The effect of the PACS on other dimensions of users' work may have been missed in this study. This effect can also be influenced by other issues such as the extent of daily PACS use and the quality of the PACS and user interface. Subsequent studies can work specifically on each of these issues. Third, in this study, we evaluated the impact of the PACS from the perspective of users. Since individuals' perspectives can be subjective, subsequent studies can use more objective methods, such as observation.

## 5. Conclusion

The results of this study showed that the PACS had a positive effect on the work dimensions of users. This system has the greatest impact on improving external communication, increasing user intention to use the system, and increasing service quality. In this study, we did not only measure the impact of the PACS on the positive aspects of users' work, such as increasing service quality, but also on the negative aspects such as complaints and pressure on the radiology department. For example, our results showed that radiology staff received more calls requesting information regarding PACS functions and services. Although different PACSs may have common advantages, several studies need to be done to identify the negative impacts of the PACS. However, in this study, radiologists have a greater understanding of the benefits of the PACS than physicians, results showed that the PACS has benefits for all user groups, and the users believe that this system has a positive impact on various dimensions of their work. This study evaluated the impact of the PACS from the users' point of view and showed that this system can positively affect five dimensions of users' work (external communication, service quality, daily routine, personal intention to use the PACS, and complaints on users). Other studies can consider other issues such as the extent of daily PACS use and the quality of the PACS and user interface.

## Figures and Tables

**Table 1:** Participants' demographics and their relationship with the impact of the PACS.

Demographic information		Frequency (%)	Mean	SD	*p*
Gender	Female	56 (77.8)	3.79	0.53	0.177
	Male	15 (2.08)	3.51	0.50	

Age (years)	Under 30	24 (33.8)	3.92	0.52	0.001
	30–39	26 (36.6)	3.84	0.46	
	40–49	16 (22.5)	3.33	0.50	
	50 or older	5 (7)	3.41	0.27	

Computer knowledge	Novice	4 (5.6)	3.28	0.34	0.11
	Average	37 (51.4)	3.85	0.38	
	Advanced	25 (34.7)	3.63	0.70	
	Expert	6 (8.3)	3.66	0.42	

Training PACS	Group training	47 (65.3)	3.80	0.54	0.037
	Individual training	14 (19.4)	3.82	0.42	
	Web-based tutorial training	4 (5.6)	3.21	0.11	
	No training	7 (9.7)	3.37	0.55	

Work experience (years)	Less than five	23 (31.9)	4.00	0.48	0.001
	5–10	2 (2.8)	3.57	0.51	
	11–15	4 (5.6)	4.07	0.49	
	More than 15	13 (18.1)	3.83	0.42	

Experience using the PACS (years)	Less than 2	34 (47.2)	3.78	0.45	0.058
	2–5	26 (36.6)	3.81	0.59	
	6–10	8 (11.3)	3.42	0.50	
	More than ten	3 (4.2)	3.13	0.40	

Job	Radiologist	9 (12.5)	4.05	0.56	<0.001
	Radiology staff	16 (22.2)	4.07	0.39	
	Physician	33 (45.8)	3.48	0.49	
	Ward's head	14 (19.4)	3.71	0.44	

**Table 2:** Pearson's correlation between study dimensions.

External communication	External communication				
Service quality	*r* = 0.631	Service quality			
	*P* < 0.001	

User intention to use the PACS	*r* = −0.88	*r* = 0.104	User intention to use the PACS		
	*P*=0.462	*P*=0.383	

Daily routine	*r* = 0.36	*r* = 0.265	*r* = 0.203	Daily routine	
	*P*=0.002	*P*=0.025	*P*=0.088	

Complaints on users	*r* = 0.021	*r* = 0.98	*r* = 0.166	*r* = 0.403	Complaints on users
	*P*=0.841	*P*=0.414	*P*=0.164	*P* < 0.001	

## Data Availability

The data used in this study are available from the corresponding author upon request.
